# Development and Functional Characterization of Fetal Lung Organoids

**DOI:** 10.3389/fmed.2021.678438

**Published:** 2021-09-06

**Authors:** Mandy Laube, Soeren Pietsch, Thomas Pannicke, Ulrich H. Thome, Claire Fabian

**Affiliations:** ^1^Division of Neonatology, Department of Paediatrics, Center for Paediatric Research Leipzig, University of Leipzig, Leipzig, Germany; ^2^Department of Vaccines and Infection Models, Fraunhofer Institute for Cell Therapy and Immunology, Leipzig, Germany

**Keywords:** lung, 3D culture, organoids, patch clamp, fetal development, model system

## Abstract

Preterm infants frequently suffer from pulmonary complications due to a physiological and structural lung immaturity resulting in significant morbidity and mortality. Novel *in vitro* and *in vivo* models are required to study the underlying mechanisms of late lung maturation and to facilitate the development of new therapeutic strategies. Organoids recapitulate essential aspects of structural organization and possibly organ function, and can be used to model developmental and disease processes. We aimed at generating fetal lung organoids (LOs) and to functionally characterize this *in vitro* model in comparison to primary lung epithelial cells and lung explants *ex vivo*. LOs were generated with alveolar and endothelial cells from fetal rat lung tissue, using a Matrigel-gradient and air-liquid-interface culture conditions. Immunocytochemical analysis showed that the LOs consisted of polarized epithelial cell adhesion molecule (EpCAM)-positive cells with the apical membrane compartment facing the organoid lumen. Expression of the alveolar type 2 cell marker, RT2-70, and the Club cell marker, CC-10, were observed. Na^+^ transporter and surfactant protein mRNA expression were detected in the LOs. First time patch clamp analyses demonstrated the presence of several ion channels with specific electrophysiological properties, comparable to vital lung slices. Furthermore, the responsiveness of LOs to glucocorticoids was demonstrated. Finally, maturation of LOs induced by mesenchymal stem cells confirmed the convenience of the model to test and establish novel therapeutic strategies. The results showed that fetal LOs replicate key biological lung functions essential for lung maturation and therefore constitute a suitable *in vitro* model system to study lung development and related diseases.

## Introduction

The study of fetal lung development is a challenging task. Any model system needs to reflect the biological properties such as morphology and function, while also being reproducible and, if possible, broadly accessible. Although many basic properties like cell morphology or protein localization can be modeled using classic 2-dimensional (2D) cell culture with cell lines or primary cells, their reflection of biological functions is often limited. The situation is further complicated by the highly complex nature of the pulmonary system with uniquely specialized cells. The lung is the central organ of the respiratory system, providing barrier function to facilitate oxygen delivery and carbon dioxide elimination. Severe clinical consequences can arise from a disruption of fetal lung development. Especially in preterm infants, pulmonary complications are common due to a physiological and structural lung immaturity leading to significant morbidity and mortality. Impaired perinatal transition from the liquid-filled to air-breathing lungs can result in respiratory distress syndrome (RDS), and possibly subsequent development of bronchopulmonary dysplasia (BPD). The pathology of BPD is based on an arrested lung development, including a reduced alveolar surface area and alveolar number, as well as impaired functions down to a cellular level. Functional disturbances of RDS mainly arise from a lack of differentiated alveolar type 2 (ATII) cells that are involved in surfactant synthesis and pulmonary fluid homeostasis. Alveolar fluid clearance (AFC) enables perinatal lung transition to air breathing that is accomplished by active Na^+^ transport across the alveolar epithelium driven by epithelial Na^+^ channels (ENaC). Importantly, ENaC expression is reduced in the preterm lungs (1), compromising AFC. These pathognomonic features must be reflected in model systems of fetal lung development. Animal models improved the understanding of lung development as well as the pathogenic mechanisms leading to RDS and BDP. Rats are a widely used animal model to reproduce the histopathology of human preterm infants with BPD. Exposing newborn rats to hyperoxia induces lung structural and functional impairment, accompanied by high levels of pulmonary inflammation (2). Thereby the critical relationship between oxygen toxicity and mechanical ventilation with lung injury and BPD development was demonstrated. However, rodent models are mainly used as endpoint models in which tissue damage and functional alterations are determined after sacrifice, preventing analysis of the disease course. Furthermore, newborn rodents are viable at birth and do not exhibit lung immaturity, which is of central importance in human BPD. Besides, respiratory distress in preterm infants is multifactorial, including congenital infection, growth restriction and placental dysfunction, and most preterm infants were exposed to clinical interventions like antenatal corticosteroids. This is not reflected in most animal models possibly leading to different responses to pathogenic challenges as well as therapeutic strategies. Therefore, novel model systems are required to study the underlying mechanisms of late lung maturation and to facilitate the development of novel therapeutic approaches. Moreover, any *in vitro* and *in vivo* model should replicate key features of lung development and maturation, and allow for a qualitative, quantitative, and most importantly, functional analysis. Immortalized lung cell lines were commonly used to determine gene expression and signaling pathways, but due to their immortalized nature, their differentiation capacity is limited. Instead, primary fetal distal lung epithelial (FDLE) cells represent a widely studied *in vitro* model due to their ability to differentiate into polarized and functional epithelia (3). FDLE cells are derived from fetal rat pups 24–48 h prior to birth. Studies showed that fetal rat pups born 24 h prior to term birth experience respiratory distress due to structural and functional lung immaturity (4). Na^+^ transport as well as the expression and secretion of surfactant proteins were studied in FDLE cells before. Furthermore, FDLE cells enable analysis of sex-specific differences between male and female cells (5), and can be used for co-culture with other primary lung cell types. On the other hand, FDLE cells are limited in their lifetime and offer only a short time window for experiments of 2–3 days.

Lung organoids (LOs) offer the chance to bridge the gap between conventional *in vitro* and *in vivo* models. Organoids are self-organizing 3D structures that can be grown from stem cells or defined tissue-specific progenitor cells (6). They supposedly recapitulate the organs' structural organization, with multiple specialized cells, and function, although actual organ-like function has yet to be determined. The first 3D lung cell cultures, called organoids or “mass cultures,” were generated using a crude cell mix derived from digested fetal lung tissue. This cell suspension consisted of epithelial, endothelial, mesenchymal and hematopoietic cells (7). By culturing the cell suspension at the air-liquid-interface (ALI) on a floating membrane filter, differentiation into mature ATII cells as well as connective tissue formation was achieved (7). This demonstrated the ability of fetal lung cells to self-organize in co-culture with other cells types and that ALI culture represents an important differentiation signal. However, lack of 3D growth limited the use of these early organoid cultures. The absence of a supporting structure, like hydrogels or a scaffold, resulted in a wide-stretched, multilayered and heterogeneous cell aggregation instead of the sophisticated 3D structures seen today. In addition, culture time was limited to a few days before mesenchymal cells overgrew and destroyed the epithelial cell structures. The culture of fetal and adult ATII cells on basement membrane extracts from Engelbreth-Holm-Swarm (EHS) tumor tissue (also known as matrix gel or Matrigel™) led to the establishment of organotypic cell cultures. ATII cell culture on polystyrol resulted in a loss of lamellar bodies, induced cell flattening and cell proliferation, while culture with EHS gels prevented the loss of lamellar bodies and the cells retained their cuboidal morphology (8, 9). These studies showed the importance of the biophysical environment, including the extracellular matrix (ECM) as well as the interface to air, to generate relevant *in vitro* lung models. The use of isolated and defined cell populations and the co-culture of lung epithelial cells with other lung-derived cell types like endothelial cells (10), mesenchymal stem cells (11), and fibroblasts (12), led to the development of self-organizing LOs reflecting morphological and cellular compositions of bronchial and alveolar tissue.

Our study focused on the generation of a biological relevant model system of fetal lung development that can be easily reproduced by other scientists. Furthermore, functional analyses demonstrated the opportunities this model offers for a variety of studies, including gene expression and patch clamp analyses. Fetal LOs were generated using FDLE and lung endothelial cells from fetal rat lungs. This allows a concise validation of the advantages and limitations of LOs as a model system for fetal lung development in comparison to the existing *in vitro* and *in vivo* studies done with rats.

## Materials and Methods

### Cell Isolation

Sprague-Dawley rats (RGD Cat# 70508, RRID:RGD_70508) were obtained from the Medical Experimental Center (MEZ) of Leipzig University. Animals were kept in rooms with a 12 h light-dark cycle, constant temperature (22°C) and humidity (55%). Food and water were supplied *ad libitum*. At gestational day E20-21 (term E22) pregnant rats were anesthetized by CO_2_ inhalation and euthanized by Pentobarbital injection. All experimental procedures were approved by the institutional review board (Landesdirektion Leipzig, permit number: T23/15). Fetal lungs were mechanically dissociated with razor blades. The resulting cell suspension was enzymatically digested with trypsin (0.125%, Fisher Scientific, Schwerte, Germany) and DNase (0.4 mg/mL, CellSystems, Troisdorf, Germany) in Hanks' Balanced Salt solution (HBSS, Fisher Scientific) for 10 min at 37°C, followed by MEM containing collagenase (0.1%, CellSystems) and DNase for 15 min at 37°C. FDLE cells, a model of fetal ATII cells, were isolated by plating the crude lung cell mix twice for 1.5 h to remove adjacent lung fibroblasts followed by differential centrifugation (3, 13). The supernatant contained FDLE cells with >95% purity (3). For the isolation of CD31^+^ endothelial cells, fetal lungs were digested using the multi tissue dissociation kit II according to the manufacturer's recommendations, followed by Magnetic Activated Cell Sorting (MACS, GentleMACS Octo) using CD31^+^ beads (all by Miltenyi Biotech, Bergisch Gladbach, Germany). The obtained cell mix was strained with a 70 μm filter (Miltenyi). Resuspension and all subsequent labeling and washing steps were done using 0.5% BSA/OptiMEM (Fisher Scientific). The cells were incubated with an anti-CD31-PE antibody (1:50, #REA396, Miltenyi) for 10 min at 4°C, followed by incubation with anti-PE magnetic beads (20 μl/10^7^ cells) for 15 min at 4°C. Up to 10^8^ cells were loaded on a LS Column (Miltenyi), inserted in a QuadroMACS™ Separator (Miltenyi) and passed twice over the same column to enrich CD31^+^ cells. To obtain a homogenous CD31^+^ cell population, two rounds of subsequent antibody-mediated cell sorting were required. The first step enriched the CD31^+^ cells to ~60–80% and the second step led to a purity of >90%. Enrichment was controlled by flow cytometry (BDAccuri, BD biosciences, San Jose, CA, USA). CD31^+^ cells were cultured on gelatin-coated flasks with Endothelial Cell Growth Supplement (ECGS) medium containing DMEM (high glucose, GlutaMAX™, Fisher Scientific), 20% FCS (Biochrom, Berlin, Germany), 15 mM HEPES (Merck, Darmstadt, Germany), Heparin (100 μg/mL, Merck), ECGS (50 μg/mL, Corning, Corning, NY, USA), penicillin (100 units/mL, Fisher Scientific), streptomycin (100 μg/mL, Fisher Scientific), and amphotericin B (0.25 μg/mL, Fisher Scientific). After ~2 weeks in culture, confluent CD31^+^ cells were used for LO generation up to passage 3 (Supplementary Figure 1). Subculture was done using TripLE™ Express (Fisher Scientific). A tube formation assay was done to determine the ability of CD31^+^ cells to form tube-like structures to verify their endothelial cell character. The tube formation assay was done by plating CD31^+^ cells at 5 × 10^4^ cells on a Matrigel-coated well of a 24-well-plate in ECGS medium. Cell morphology was analyzed after 22 h (Supplementary Figure 1).

### 3D Culture

Transparent permeable transwell inserts (ThinCert, #662610, surface area 33.6 mm^2^, Greiner Bio-One, Frickenhausen, Germany) were first coated with growth-factor reduced (GFR) Matrigel (3 mg/mL, #356230, Corning) at 37°C. Matrigel GFR was dissolved in ice-cold LO medium (LO-Med) consisting of DMEM/F12 (#31330095, Fisher Scientific), 10% FCS, 1% insulin-transferrin-selenium-ethanolamine (ITS-X, #51500056, Fisher Scientific), 1 mM HEPES (#A1069,0250, AppliChem, Darmstadt, Germany), penicillin (100 units/mL), streptomycin (100 μg/mL) and amphotericin B (0.25 μg/mL). Freshly isolated FDLE cells (1.5 × 10^5^ per well) were mixed with CD31^+^ cells (0.375 × 10^5^ per well), constituting an epithelial to endothelial cell ratio of 1:0.25. The defined cell mix was combined with Matrigel GFR (0.4 mg/mL in LO-Med) and transferred to the coated inserts. The lower compartment of the transwell insert was filled with LO-Med, while the upper compartment containing the cells in Matrigel was not submerged in medium enabling air-liquid interface (ALI) conditions. LOs were cultured at 37°C with medium exchange every 2 days. Live cell imaging was done using a microscope (CKX41, Olympus, Hamburg, Germany) with a temperature controller (ibidi, Graefelfing, Germany) and a CO_2_ controller (The Brick Gas Mixer, Life Imaging Services, Basel, Switzerland) during the first day of FDLE and CD31^+^ co-culture under submerged culture conditions to determine their initial self-organization. To analyze the effect of specific stimulating agents LO-Med was supplemented with dexamethasone (100 nM, Merck). Furthermore, the effect of mesenchymal stem cell-conditioned medium (MSC-CM) on LOs was determined after the organoids reached 15 days *in vitro* (div). The organoids were further cultured for 4 additional days in MSC-CM or the corresponding medium used for the culture of MSCs, which consisted of DMEM (low glucose, GlutaMAX™, Fisher Scientific) and 2% FCS. To subculture LOs or obtain them for further analyses, cell recovery solution (#354253, Corning) was used. To this end, all inserts were incubated on ice for ~2 h with ice cold cell recovery solution. Subsequently, the cell recovery solution was replaced by sterile 10% BSA (#8076.2, Carl Roth, Karlsruhe, Germany) in PBS. The digested Matrigel solution was centrifuged at 300 × g for 5 min, sedimented LOs were resuspended, washed twice in PBS and used for patch clamp analyses or fixed with 2% formaldehyde (#11586711, Fisher Scientific) in PBS for 20 min at room temperature followed by embedding in Tissue-Tek™ O.C.T. (#4583, Weckert, Kitzingen, Germany) for immunofluorescence staining. LO compactness was defined as the cellular area in proportion to the whole organoid area using Image J version 1.53c (ImageJ, RRID:SCR_003070) as published before (14). Compactness, also known as solidity, includes the packing density and the intercellular space cavities thereby indicating internal cellularity and external branching.

### Immunofluorescence

Characterization of LOs was carried out with immunofluorescence staining of cryotome sections (5 μm). LO slices were washed with PBS and then treated with 5% BSA/PBS containing 0.5% Triton-X 100 (Merck) for 1 h at room temperature. Afterwards, the slices were incubated at 4°C overnight with the respective primary antibody, diluted in 5% BSA/PBS. LO slices were incubated with rabbit-anti-epithelial cell adhesion molecule (EpCAM) primary antibody (1:50; # ab71916, Abcam, Cambridge, UK, RRID:AB_1603782), mouse-anti-RT1-40 (1:150, #TB-11ART1-40, Terrace Biotech, San Francisco, CA, USA) for staining of ATI cells (15), rabbit-anti-aquaporin 5 (Aqp 5, 1:100, #178615, Merck, RRID:AB_211472), mouse-anti-RT2-70 (1:150, #TB-44ART2-70, Terrace Biotech) to detect ATII cells (16), rabbit-anti-Club cell secretary protein (CC-10, 1:100, #ab40873, Abcam, RRID:AB_778766), and rabbit-anti-Ki-67 (1:500, #9129, Cell Signaling Technology, Danvers, MA, USA, RRID:AB_2687446). Control slices were treated with 5% BSA/PBS without the primary antibody. The secondary antibody NL-493 (1:200; R&D Systems, Boston, USA, RRID:AB_663764) was used for primary rabbit IgG antibodies and NL-637 (1:200; R&D Systems, RRID:AB_663771) was used for primary mouse IgG antibodies. Nuclei were stained with DAPI (1 μg/ml, #D8417-1MG, Merck). For Live-Dead staining LOs were resuspended in fresh LO-Med containing 2 mg/mL Matrigel and plated on a glass bottom dish (#81218-200, ibidi). After 3 days, LO-Med was replaced by a solution containing Calcein-AM (5 μM, #sc-203865, Santa Cruz) and propidium iodide (PI, 1 μg/mL, #CN74.1, Carl Roth). Staining of actin filaments was performed with FITC-labeled phalloidin (2.5 μg/mL, #P1951, Merck, RRID:AB_2315148). All sections were covered with ProLong™ Glass Antifade Mountant (#P36980, Fischer Scientific). For image capturing a confocal laser-scanning microscope (LSM710, Laser: Diode 405, Argon 488, Helium-Neon 543; Objective: Plan- Apochromat 20 x, Zeiss, Goettingen, Germany) was used. Area and fluorescence calculation were done with Image J. Calculation of cell numbers was done by manual counting. Depending on the used antibodies, either whole positively stained cells (EpCAM) or in case of apical markers, cells with an adjacent positive staining (RT2-70), were counted as positive.

### Gene Expression Analyses

RNA isolation was done at 15 div using the Purelink RNA Mini Kit (Fisher Scientific) according to the manufacturer's instructions. Reverse transcription was carried out using the Maxima H Minus First Strand cDNA Synthesis Kit with dsDNase (Fisher Scientific). Real-time quantitative PCR (RT-qPCR) was done in the CFX 96 Real-Time PCR Detection System (Bio-Rad, Munich, Germany) using the SYBR Select Master Mix (Fisher Scientific) and gene-specific primers listed in Table 1. A serial dilution of target-specific plasmid DNA was used for absolute quantification. Molecule concentrations were then normalized to a reference gene encoding for the mitochondrial ribosomal protein S18a (*Mrps18a*). Constant expression of *Mrps18a* was confirmed against other common reference genes. Using the relative standard curve method mRNA levels were calculated and expressed as relative fold change of the respective control. Melting curves and gel electrophoresis of PCR products were routinely performed to control the specificity of the PCR reaction.

**Table 1:** Primer sequences.

**Gene**	**Primer (forward, 5′-3′)**	**Primer (reverse, 5′-3′)**
***α-ENaC*** NM_031548.2	TTCTGGGCGGTGCTGTGGCT	GCGTCTGCTCCGTGATGCGG
***β-ENaC*** NM_012648.1	TGCAGGCCCAATGCCGAGGT	GGGCTCTGTGCCCTGGCTCT
***γ-ENaC*** NM_017046.1	CACGCCAGCCGTGACCCTTC	CTCGGGACACCACGATGCGG
***Na,K-ATPases-α**_**1**_* NM_012504.1	GGACGAGACAAGTATGAGCCCGC	CATGGAGAAGCCACCGAACAGC
***Na,K-ATPases-β**_**1**_* NM_013113.2	GCGCAGCACTCGCTTTCCCT	GGGCCACACGGTCCTGGTACG
***CFTR*** NM 031506.1	GCCTTCGCTGGTTGCACAGTAGTC	GCTTCTCCAGCACCCAGCACTAGA
***Mrps18a*** NM_198756.2	GCGACCGGCTGGTTATGGCT	GGGCACTGGCCTGAGGGATTAG
***Sftpa*** (17) NM_001270647.1	CCTCTTCTTGACTGTTGTCGCTGG	GCTGAGGACTCCCATTGTTTGCAG
***Sftpb*** (17) NM_138842.1	GGAGCTAATGACCTGTGCCAAGAG	CTGGCCCTGGAAGTAGTCGATAAC
***Sftpc*** (17) NM_017342.2	GATGGAGAGCCCACCGGATTACTC	GAACGATGCCAGTGGAGCCAATAG

### Patch Clamp Analyses

Patch clamp studies were performed at 15 div. LOs were transferred to the recording chamber in a bath on the stage of a microscope (BX61WI, Olympus), which was filled with a solution containing (mM): 135 KCl, 2 MgCl_2_, 6 NaCl, 5.5 Glucose, 10 HEPES (pH 7.4). Cell attached currents were recorded with an EPC10 patch clamp amplifier (Heka Elektronik, Lambrecht, Germany). A standard personal computer running Patchmaster software (Heka, Patchmaster, RRID:SCR_000034) controlled the EPC10 and stored the current tracings. Patch pipettes were pulled from borosilicate capillaries with 1.5 mm outer diameter and 0.86 mm inner diameter (Science Products, #GB150-8P) using a P2000 laser puller (Sutter, Novato, CA). The pipettes were filled with a solution containing (mM): 140 NaCl, 5 KCl, 1 MgCl_2_, 1.8 CaCl_2_, 5.5 Glucose, 10 HEPES (pH 7.4), resulting in a tip resistance between 4 and 6 MΩ. ATII cells were identified using Lysotracker (1 μM, LysoTracker Green DND-26, Fisher Scientific), which selectively accumulates in their lamellar bodies (18). After forming a gigaohm seal, currents were recorded at membrane potentials between −100 and +100 mV in 10 mV increments, filtered at 2 kHz and sampled at 10 kHz. Cell attached recordings were analyzed with Fitmaster software (Heka, Fitmaster, RRID:SCR_016233). Voltages are given as the negative of the patch pipette potential, which represents the shift of the patch potential from the resting potential. Negative potentials represent hyperpolarization, and positive potentials represent depolarization of the cell membrane away from the resting potential. Highly selective cation (HSC) channels and non-selective cation (NSC) channels were identified by characteristic channel kinetics and the current-voltage relationship for the channel.

### Ussing Chamber Measurements

Ussing chamber measurements of FDLE cells were performed 4 days after cell isolation, as previously reported (5). Only monolayers with a transepithelial resistance (*R*_te_) exceeding 300 Ω·cm^2^ were included in the analyses. Electrophysiological solutions consisted of: 145 mM Na^+^, 5 mM K^+^, 1.2 mM Ca^2+^, 1.2 mM Mg^2+^, 125 mM Cl^−^, 25 mM ${\text{HCO}}_{3}^{-}$, 3.3 mM H_2_${\text{PO}}_{4}^{-}$, and 0.8 mM ${\text{HPO}}_{4}^{2-}$ (pH 7.4). For the basolateral solution, 10 mM glucose was used, while 10 mM mannitol was used in the apical solution. During measurements, the solutions were continuously bubbled with carbogen (5% CO_2_ and 95% O_2_). Equivalent short-circuit currents (*I*_SC_) were determined every 20 s by measuring transepithelial voltage (*V*_te_) and *R*_te_ with a transepithelial current clamp (Physiologic instruments, San Diego, CA) and calculating the quotient *I*_SC_ = *V*_te_/*R*_te_. After the *I*_SC_ reached a stable plateau (*I*_base_), amiloride (10 μM, # A7410, Sigma-Aldrich) was applied to the apical chamber to assess the amiloride-sensitive Δ*I*_SC_ (Δ*I*_amil_). The current reduction induced by amiloride (Δ*I*_amil_) was used as a measure of ENaC activity. Amiloride was dissolved in water.

### Isolation of Human Mesenchymal Stem Cells

The study was approved by the ethical board of the medical faculty of Leipzig University. The umbilical cord tissue was collected after delivery from human newborns whose mothers granted informed consent. MSC isolation and characterization are described elsewhere (19). MSC-CM was produced by incubating the culture medium with MSCs for 72 h, followed by sterile filtration.

### Statistical Analyses

Differences between two groups were analyzed with the unpaired *T*-test or the Mann-Whitney test. A probability of *p* < 0.05 was considered significant for all statistical analyses. Statistical analysis was performed with GraphPad Prism software (GraphPad Software, La Jolla, CA, USA, RRID:SCR_002798).

## Results

### Development of Fetal LOs

**Figure 1:**
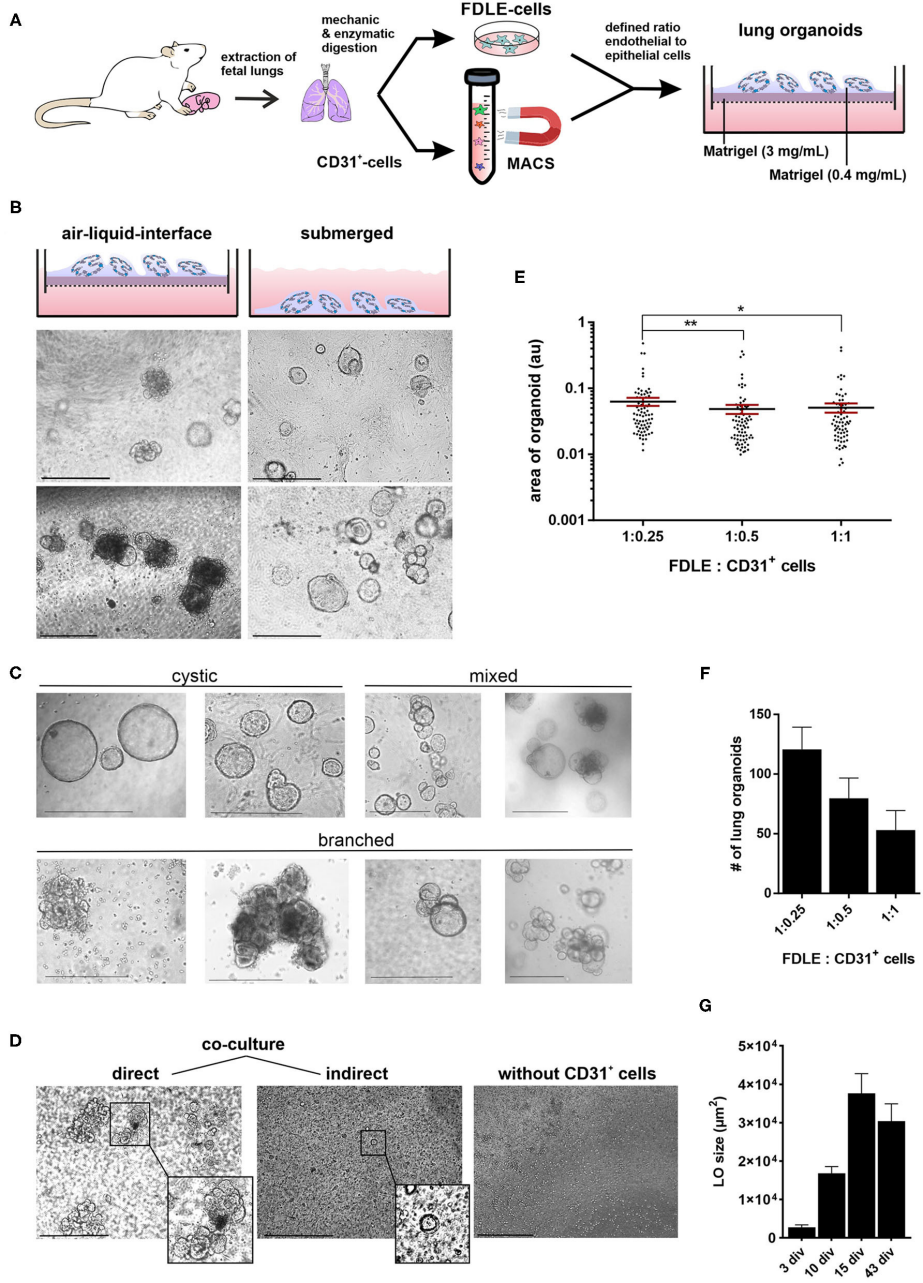
Generation of fetal LOs. **(A)** Schematic illustration of LO generation. CD31^+^ cells were mixed with freshly isolated FDLE cells and used for LO generation. **(B)** LO formation was compared between ALI culture, in which LOs were cultured on permeable inserts exposed to air, and submerged culture without air exposure. ALI conditions resulted in more divers and complex LOs of branched and cystic morphology, while the submerged culture condition mainly led to the formation of cystic LOs (pictures were taken at 15 div). **(C)** Exemplary demonstration of different morphologies (cystic, branched or a mixed appearance) observed during LO culture. **(D)** The 3D assembly was enhanced by direct interaction of FDLE cells with CD31^+^ cells (3 div), while indirect co-culture delayed LO formation (3 div). Furthermore, without CD31^+^ cells no LO formation was observed (4 div). **(E)** Different cell ratios were tested (FDLE to CD31^+^ cells of 1:1, 1:0.5, and 1:0.25). Data of LO area are displayed in a scatter dot plot with mean (horizontal line) ± SEM. Increasing the number of CD31^+^ cells decreased LO area (*n* = 70–75; ^**^p < 0.01; ^*^p < 0.05 by *T*-test). **(F)** Numbers of LOs are displayed as mean + SEM. Increasing the number of CD31^+^ cells further decreased LO numbers. **(G)** LO size (μm^2^) at 3, 10, 15, and 43 div (*n* = 40). div, days *in vitro*. Scale bar: 500 μm.

The generation of LOs is schematically illustrated in Figure 1A. The isolation of CD31^+^ cells was done by antibody-mediated cell sorting using magnetic beads. This allowed analysis of cells during every step of the isolation process: the initial amount of CD31^+^ cells in the total lung cell mix, the CD31^+^ cells bound to the magnetic column as well as the non-bound cells in the flow-through. CD31^+^ cells represented 5.2 ± 2.6% (Mean ± SD; *n* = 16) of total lung cells. CD31^+^ cells were cultured for 7–14 days to increase their cell numbers. These CD31^+^ cells were combined with freshly isolated FDLE cells and used for organoid generation. LO formation was compared between Matrigel-coated permeable inserts covered with cells in Matrigel exposed to air (ALI culture) and Matrigel-containing cell suspension plated at the bottom of a well and overlaid with LO-Med (submerged culture) (Figure 1B). The ALI condition resulted in more diverse and complex LO formation of branched and cystic morphology, while the submerged culture condition mainly led to the formation of cystic LOs. In some cystic LOs, differentiated cells with beating cilia within the lumen were observed (data not shown). Figure 1C shows examples of the different morphologies observed. Cystic LOs exhibited one lumen and were mainly transparent. Branched morphology consisted of several cysts attached to each other, reminding of budding structures or even more condensed structures with opaque appearance. The discrimination between cystic and branched morphology was done according to published studies (20). Submerged cultures were not investigated further because of the morphology and presence of cilia. Testing different FDLE cell numbers demonstrated that at least 0.5 × 10^5^ cells per well were required for 3D LO formation, otherwise only 2D cell layers were observed. Furthermore, without CD31^+^ cells no 3D LOs were observed (Figure 1D). CD31^+^ cells began to align themselves in the Matrigel and formed tube-like structures within the first 24 h of culture (Supplementary Figures 1B,C). Without CD31^+^ cells, FDLE cells did not show active migration, but in co-culture cell-cell adhesive interactions between FDLE and CD31^+^ cells led to initial cell aggregates (Supplementary Figure 1C). The 3D assembly was enhanced by direct interaction of FDLE cells with CD31^+^ cells, while indirect co-culture delayed LO formation by ~1 week (Figure 1D). Different cell ratios were tested (FDLE to CD31^+^ cells of 1:1, 1:0.5, and 1:0.25). Increasing the number of CD31^+^ cells decreased LO area and number (Figures 1E,F). After 15 days in culture LOs reached mean sizes of 115.34 ± 44.82 μm (Mean ± SEM, *n* = 22). LO size increased during culture reaching a maximum at about 15 div, no further increase of LO size was observed at 43 div (Figure 1G). Prolonged LO culture led to overgrowth of a cell layer after ~1 month that consumed all medium and impaired organoid growth. Thus, splitting of LO cultures was necessary, which enabled further subculture, without the need to add additional CD31^+^ cells. Using fetal adjacent lung fibroblasts or human umbilical vein-derived endothelial cells (HUVECs) instead of CD31^+^ cells also resulted in the formation of 3D LOs.

### Morphological Characterization of Fetal Lung Organoids

**Figure 2:**
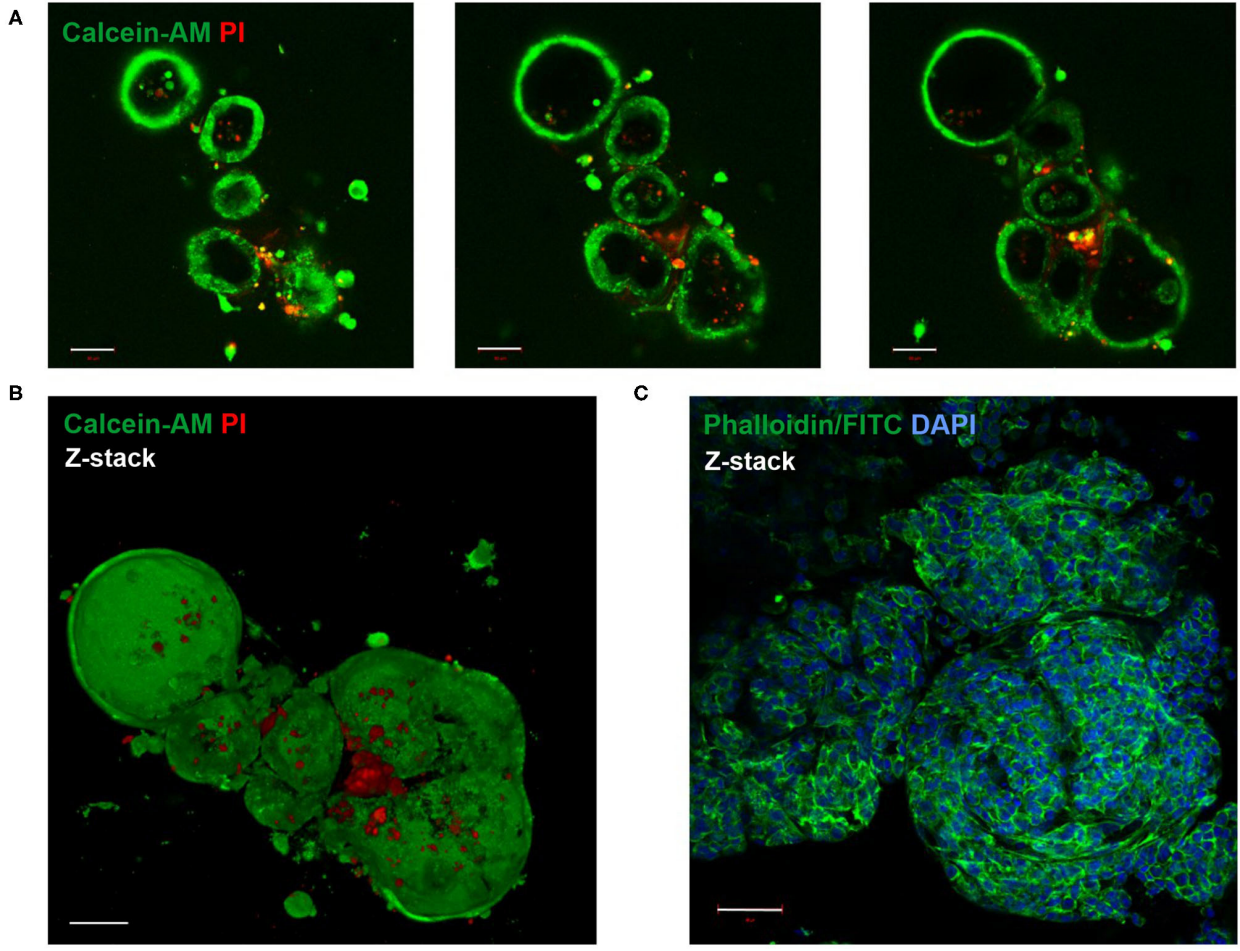
Live-Dead and F-actin staining of fetal LOs. Fluorescence Z-stack serial images were taken by confocal microscopy. The bottom-to-top distance was 58 μm with 1 μm distance intervals between images. **(A)** Live-Dead staining with Calcein-AM (green) that accumulated in living cells and propidium iodide (PI, red) that accumulated in dead cells (23 div). Images of different optical sections through the LOs (11, 22, and 30 μm distance from bottom) demonstrated that the majority of cells were alive. **(B)** Z-stack image of **(A)** showed that dead cells were mainly attached to the LOs, while the LO structures consisted of living cells. **(C)** Z-stack image of F-actin staining that showed the structural organization of the LO (17 div) by phalloidin-FITC fluorescence (green). Nuclei were stained with DAPI (blue). div, days *in vitro*. Scale bar: 50 μm.

**Figure 3:**
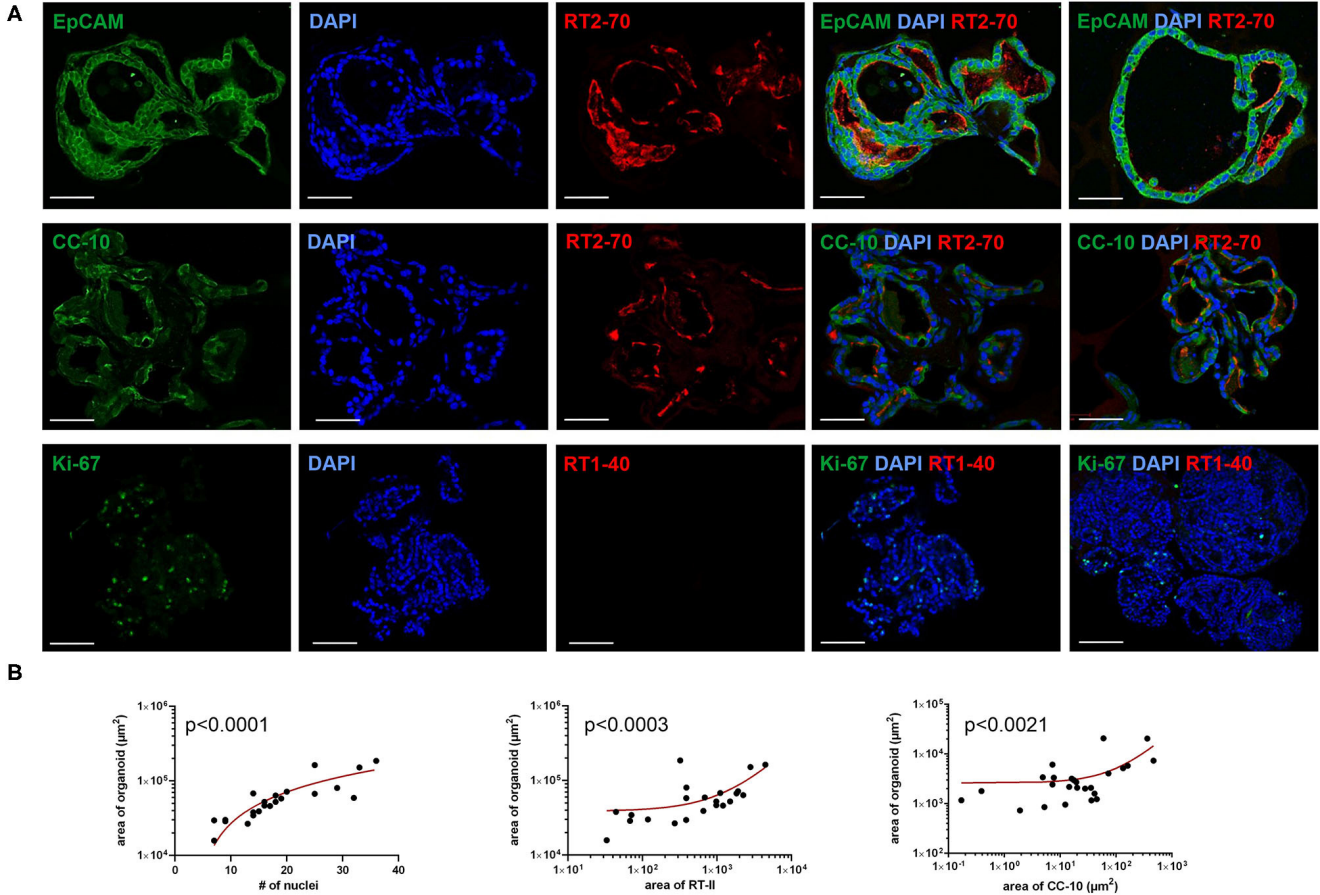
Immunofluorescence characterization of fetal LOs. Fluorescence images of LO slices (15 div) were taken by confocal microscopy. **(A)** Organoids were strongly positive for EpCAM expression. Nuclei were stained with DAPI. Epithelial cells within the LOs polarized with the apical membrane compartment facing the lumen, as shown by the luminal expression of the RT2-70 antigen. Furthermore, expression of the Club cell marker CC-10 was detected. In contrast, the ATI cell marker RT1-40 was rarely observed in the LOs. Ki-67 staining showed that only a small subset of the total cells was actively proliferating. Scale bar: 50 μm. **(B)** The area of LOs positively correlated with the number of nuclei (*n* = 21; *p* < 0.0001). The area of RT2-70^+^ cells positively correlated with LO area (*n* = 21; *p* < 0.0003). Furthermore, area of CC-10^+^ expression positively correlated with LO area (*n* = 25; *p* < 0.0021). div, days *in vitro*.

Live-Dead staining of passaged LOs showed that the majority of cells within the organoid were alive (Figures 2A,B). Staining with phalloidin-FITC was used to visualize the structural organization of filamentous actin (F-actin) with a cobblestone-like epithelial appearance of the LOs (Figure 2C). The cellular composition of the LOs consisted only of EpCAM^+^ cells without direct integration of CD31^+^ cells. We did not perform an additional staining of CD31^+^ cells in the lung organoids at 15 div, since our initial experiments testing different medium conditions (data not shown) showed that the culture condition used for lung organoids was not supporting CD31^+^ cells growth during long term culture. LOs were strongly positive for EpCAM expression (Figure 3A). Notably, epithelial cells within the LOs polarized with the apical membrane compartment facing the lumen of the organoid, as shown by the luminal expression of the RT2-70 antigen. Furthermore, expression of the Club cell marker CC-10 was detected. In contrast, the ATI cell markers, RT1-40 (T1α or podoplanin) and Aqp5 were not observed in the LOs. Notably, RT1-40 expression was also not detected in fetal rat lung slices (E21), while their pronounced and widespread expression was detected in adult rat lung tissue (Supplementary Figure 2). Ki-67 staining showed that only a small subset of the total cells was actively proliferating at 15 div.

The area of LOs positively correlated with the number of nuclei, determined by DAPI staining (Spearman r = 0.89; *p* < 0.001; Figure 3B). The positively stained area for ATII cells (RT2-70^+^) positively correlated with LO area (in μm^2^; Spearman r = 0.703; *p* < 0.001; Figure 3B), and 1.59 ± 0.37% of the LO area expressed the RT2-70 antigen, independent of organoid size. Furthermore, 1.38 ± 0.30% of total LO area was CC-10^+^, whose expression also positively correlated with organoid size (Spearman r = 0.619; *p* < 0.01; Figure 3B). Notably, the positively stained area (in%) does not reflect the actual cell number.

### Functional Characterization of Fetal Lung Organoids

**Figure 4:**
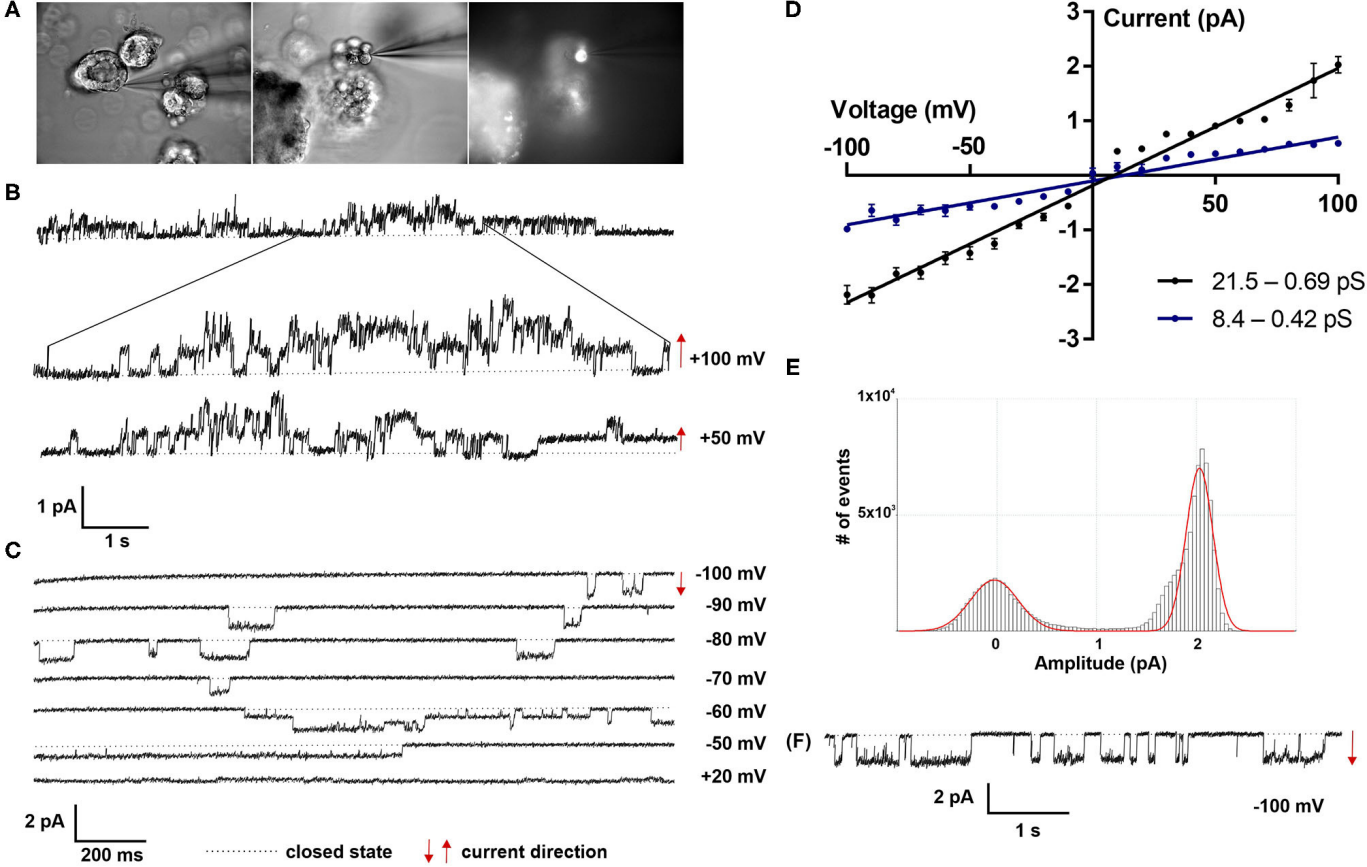
Electrophysiological characterization of fetal LOs. Patch clamp analyses of LOs at 15 div demonstrated the presence of ion channels of different conductance, open probabilities and open and closed times. **(A)** ATII cells within the LOs were identified by fluorescence after incubation with Lysotracker. **(B)** Single as well as multiple channel activities were observed at different holding potentials (+100 and +50 mV), showing exemplary outward currents as demonstrated by the upward direction of openings from the closed state. **(C)** Furthermore, inward currents were observed at different negative holding potentials (−100 to +20 mV) as shown by the downward direction of the openings from the closed state. **(D)** Plotting the current-voltage relationship identified two types of channels: a channel with a conductance of 8.4 ± 0.42 pS (slope ± SE, *n* = 18 cells) and a reversal potential of 11.3 mV (R^2^ = 0.982), and a second channel with a conductance of 21.5 ± 0.69 pS (*n* = 15 cells) that reversed at 9.2 mV (R^2^ = 0.955). **(E,F)** Amplitude histogram and current tracing of single channel openings at −100 mV. Closed state is indicated by the dotted line. The arrows indicate the current direction with ↑ outward and ↓ inward currents.

**Figure 5:**
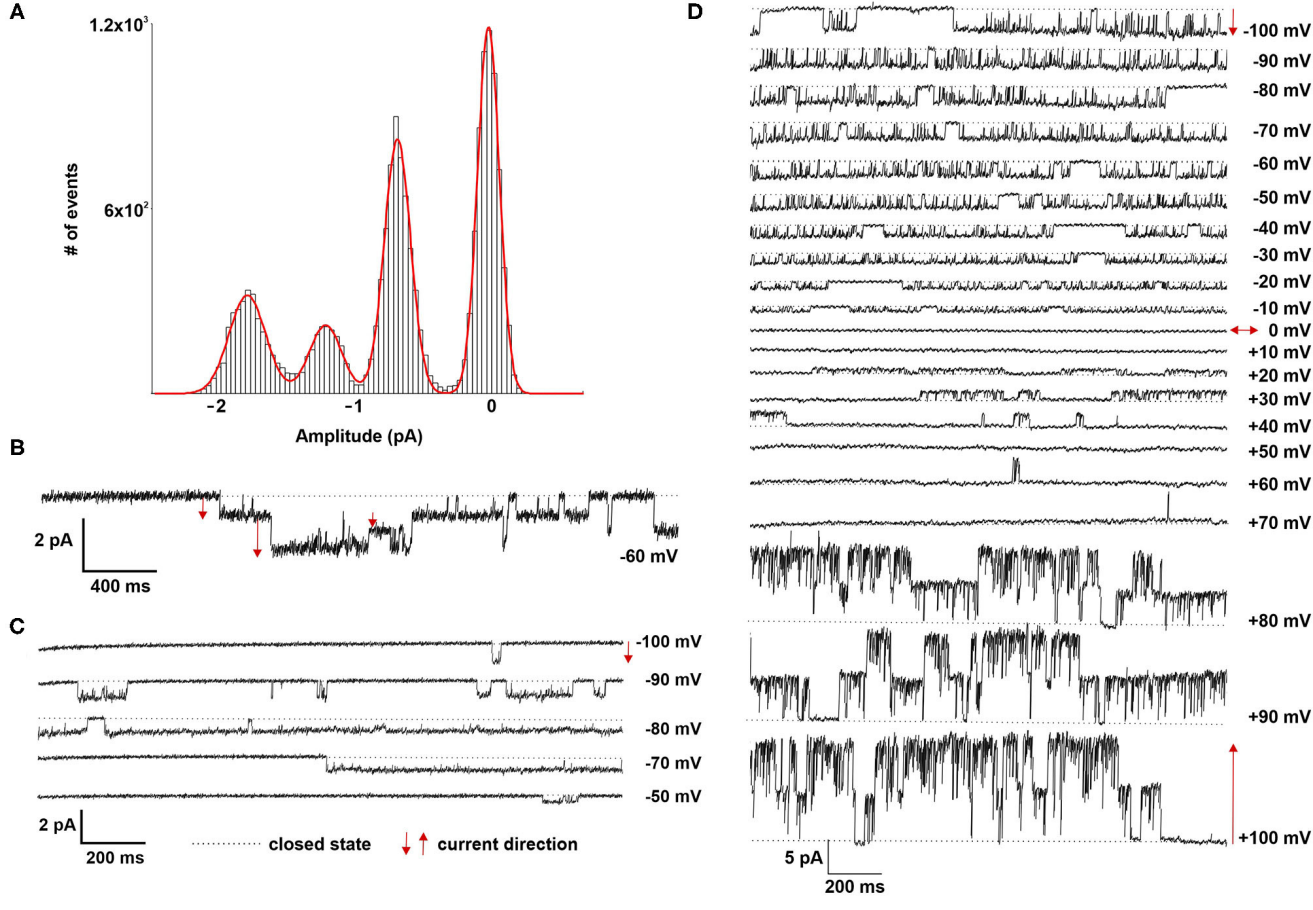
Electrophysiological characterization of fetal LOs. **(A,B)** Amplitude histogram and current tracing of multiple channel openings at −60 mV. **(C)** Different open times at different holding potentials (−100 to −50 mV) are displayed. **(D)** High variability of channel kinetics (amplitude, open and closed times) are shown for a recording with voltage steps from −100 to +100 mV and a reversal potential close to 0. The strong rectifying properties of the illustrated recording, with smaller inward currents on hyperpolarization and larger outward currents when the patch membrane was depolarized, suggests an outwardly rectifying Cl^−^ channel (ORCC). Closed state is indicated by the dotted line. The arrows indicate the current direction with ↑ outward and ↓ inward currents.

Patch clamp analyses of LOs demonstrated the presence of ion channels of different conductance, open probability and open and closed time. First, ATII cells within the LOs were identified by fluorescence staining after incubation with Lysotracker (Figure 4A). Single as well as multiple channel activities were observed at different holding potentials, showing exemplary outward currents as demonstrated by the upward direction of openings from the closed state (Figure 4B). Furthermore, inward currents were observed at different negative holding potentials as shown by the downward direction of the openings from the closed state (Figure 4C). Plotting the current-voltage relationship identified two types of channels: a channel with a slope conductance of 8.4 ± 0.42 pS (slope ± SE, *n* = 18 cells) and a reversal potential of 11.3 mV (R^2^ = 0.982), and a second channel with a conductance of 21.5 ± 0.69 pS (*n* = 15 cells) that reversed at 9.2 mV (R^2^ = 0.955) (Figure 4D). Thus, ATII cells within the LOs displayed functional epithelial Na^+^ channels with HSC and NSC channel-like transport properties. Figures 4E,F, 5A,B further show the amplitude histograms of single and multiple channel openings on hyperpolarization. Different open times at different holding potentials are displayed in Figure 5C. Finally, high variability of channel kinetics (amplitude, open and closed times) are shown for a recording with voltage steps from −100 to +100 mV and a reversal potential close to 0 (Figure 5D). According to the strong rectifying properties of the illustrated recording, with smaller inward currents on hyperpolarization (28.41 pS) and larger outward currents when the patch membrane was depolarized (82.54 pS), an outwardly rectifying Cl^−^ channel (ORCC) can be assumed.

### Reviewing the Effect of Certain Lung-Stimulating Factors

**Figure 6:**
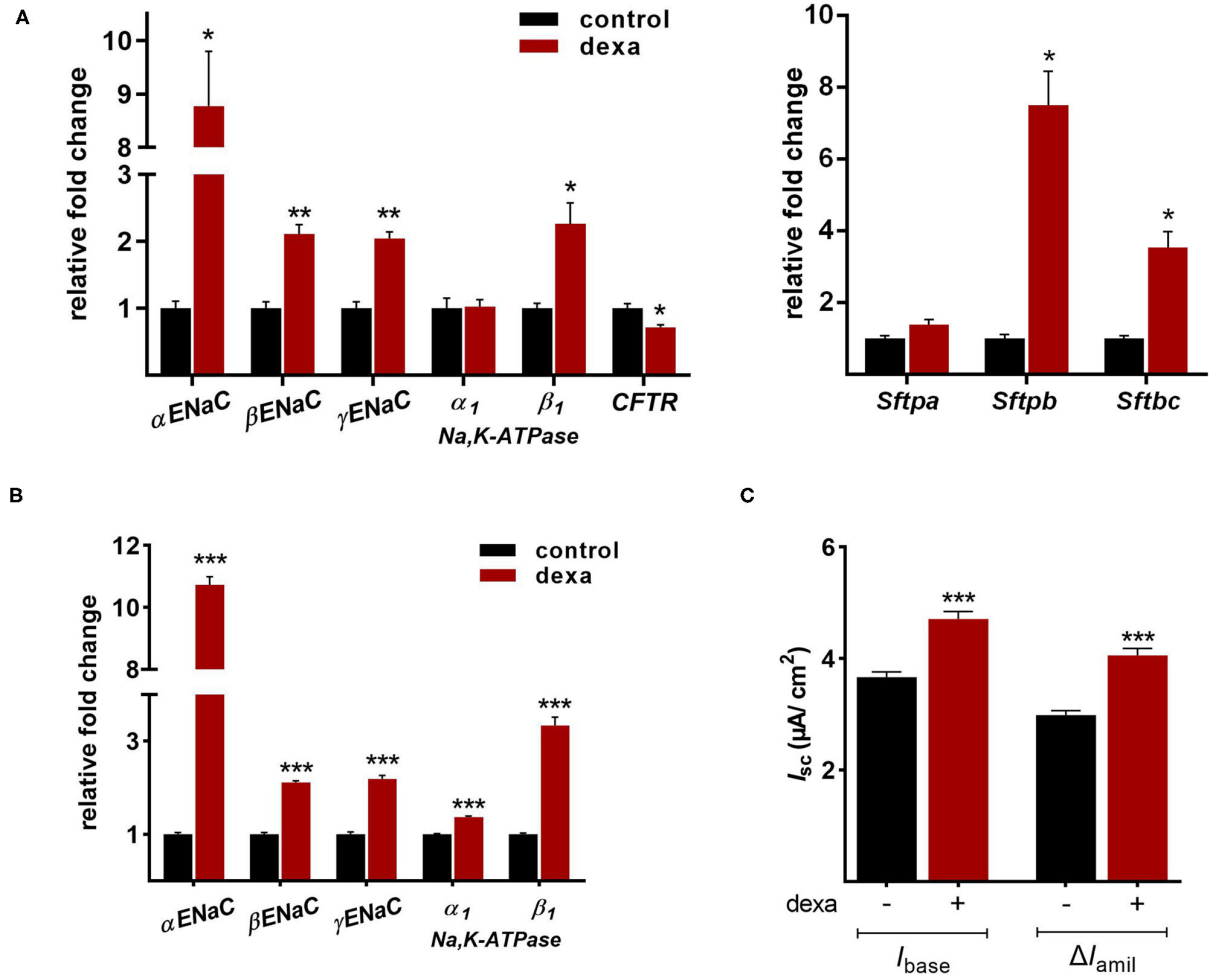
Effects of dexamethasone on fetal LOs compared to FDLE cells. Data are displayed as Mean + SEM. **(A)** Gene expression analysis of LOs (15 div) stimulated with dexamethasone (100 nM) for 48 h. Dexamethasone significantly increased mRNA expression of the *ENaC subunits* (α, β, γ) and the *Na,K-ATPase subunit-*β*1* compared to control LOs cultured without dexamethasone (*n* = 4/5; ^**^p < 0.01; ^*^p < 0.05 by *T*-test). mRNA expression of *CFTR* was reduced by dexamethasone (^*^p < 0.05 by *T*-test). Furthermore, the mRNA expression of *Sftpb* and *Sftpc* were significantly increased by dexamethasone (*n* = 4; ^*^p < 0.05 by *T*-test). **(B)** FDLE cells were also stimulated with dexamethasone (100 nM) for 48 h. In accordance to LOs, dexamethasone strongly increased the mRNA expression of the Na^+^ transporters (*n* = 12; ^***^p < 0.001 by *T*-test). **(C)** The increased mRNA expression was accompanied by an enhanced Na^+^ transport (*I*_base_) and ENaC activity (Δ*I*_amil_) in FDLE cells, as determined in Ussing chambers (*n* = 78/92; ^***^p < 0.001 by *T*-test). div, days *in vitro*.

**Figure 7:**
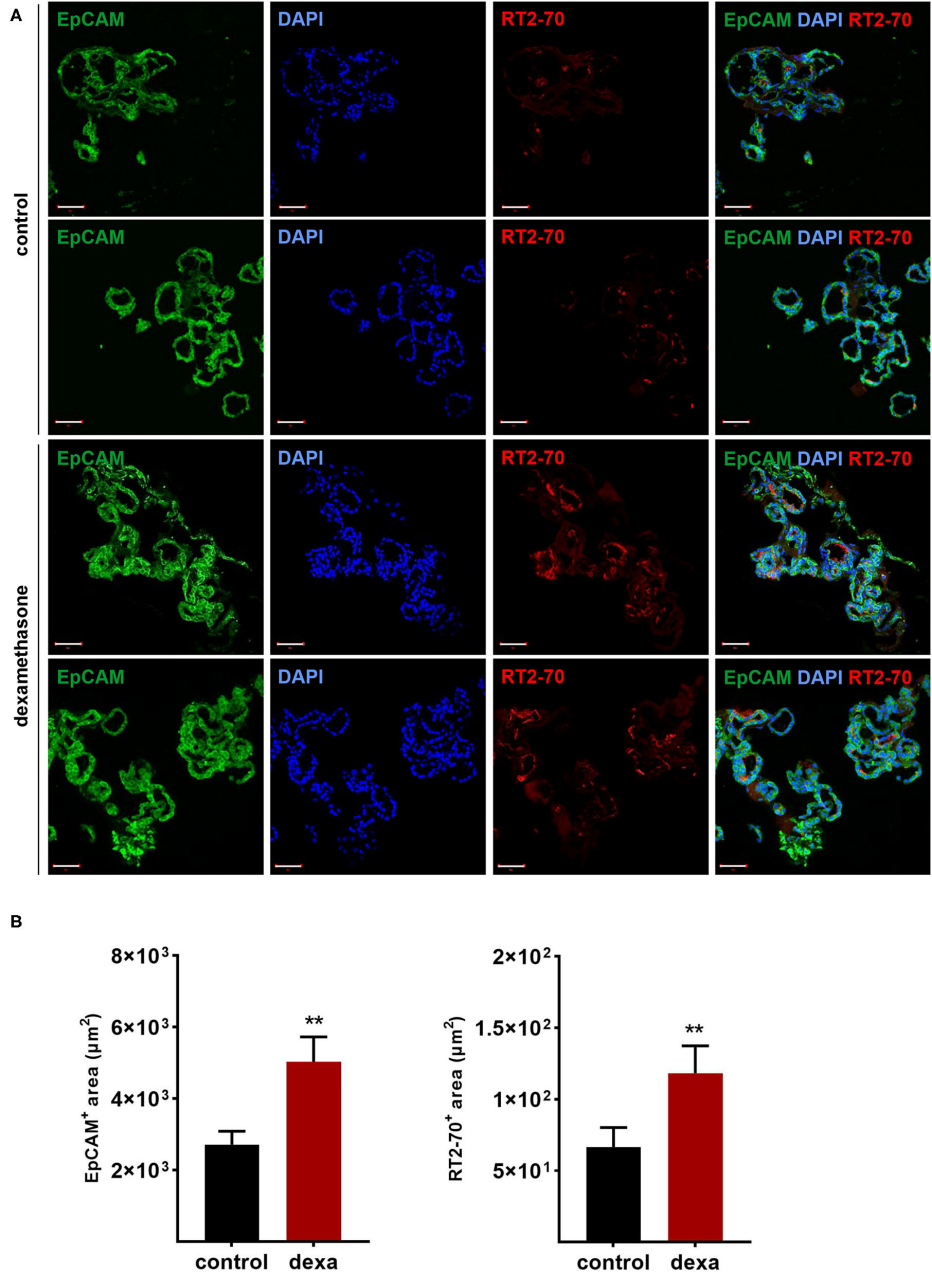
Effects of dexamethasone on fetal LOs. LOs (15 div) were stimulated with dexamethasone (100 nM) for 48 h. Fluorescence images of LO slices were taken by confocal microscopy. **(A)** Organoids were strongly positive for EpCAM expression. Nuclei were stained with DAPI. Some epithelial cells showed luminal expression of the RT2-70 antigen. Scale bar: 50 μm. **(B)** Data are displayed as Mean + SEM. The area of EpCAM^+^ area and RT2-70^+^ area was higher in LOs stimulated with dexamethasone compared to control LOs (*n* = 25; ^**^p < 0.01 by Mann-Whitney test). div, days *in vitro*.

Gene expression analysis of LOs demonstrated the mRNA expression of *ENaC* and the *Na,K-ATPase* as well as surfactant protein (*Sftp*)-A, B, and C, which exhibit essential functions in mature ATII cells. Dexamethasone is known to increase the mRNA expression of Na^+^ transporters and surfactant genes *in vitro* and *in vivo*. Thus, LOs were incubated with dexamethasone (100 nM) for 48 h, prior to RNA isolation at 15 div. Dexamethasone significantly increased mRNA expression of the *ENaC subunits* (α, β, γ) and the *Na,K-ATPase subunit-*β*1* compared to control LOs cultured without dexamethasone (*p* < 0.01, *p* < 0.05; Figure 6A). In contrast, mRNA expression of the cystic fibrosis conductance regulator (*CFTR*) was significantly reduced by dexamethasone (*p* < 0.05). Furthermore, the mRNA expression of *Sftpb* (surfactant protein B) and *Sftpc* (surfactant protein C) were significantly increased by dexamethasone in LOs (*p* < 0.01, *p* < 0.05; Figure 6A). In agreement, dexamethasone significantly increased mRNA expression of ENaC and the Na,K-ATPase in primary FDLE cells grown on permeable inserts (*p* < 0.001; Figure 6B). Furthermore, Na^+^ transport was significantly enhanced by dexamethasone as determined in Ussing chambers (Figure 6C). Dexamethasone increased *I*_base_ from 3.67 ± 0.09 μA/cm^2^ (Mean ± SEM) to 4.71 ± 0.13 μA/cm^2^ and the Δ*I*_amil_ from 2.99 ± 0.08 μA/cm^2^ to 4.06 ± 0.12 μA/cm^2^ (*p* < 0.001; Figure 6C). These results show that the fetal LOs mimic the response to dexamethasone seen in primary fetal lung epithelia and that the observed increase of mRNA expression causes an elevated transepithelial Na^+^ transport activity. Furthermore, LOs were strongly positive for EpCAM expression (Figure 7A). Notably, dexamethasone significantly increased expression of the EpCAM and RT2-70 antigen (*p* < 0.01; Figure 7B). Finally, morphology of LOs treated with dexamethasone was not altered compared to control LOs (Supplementary Figure 3).

**Figure 8:**
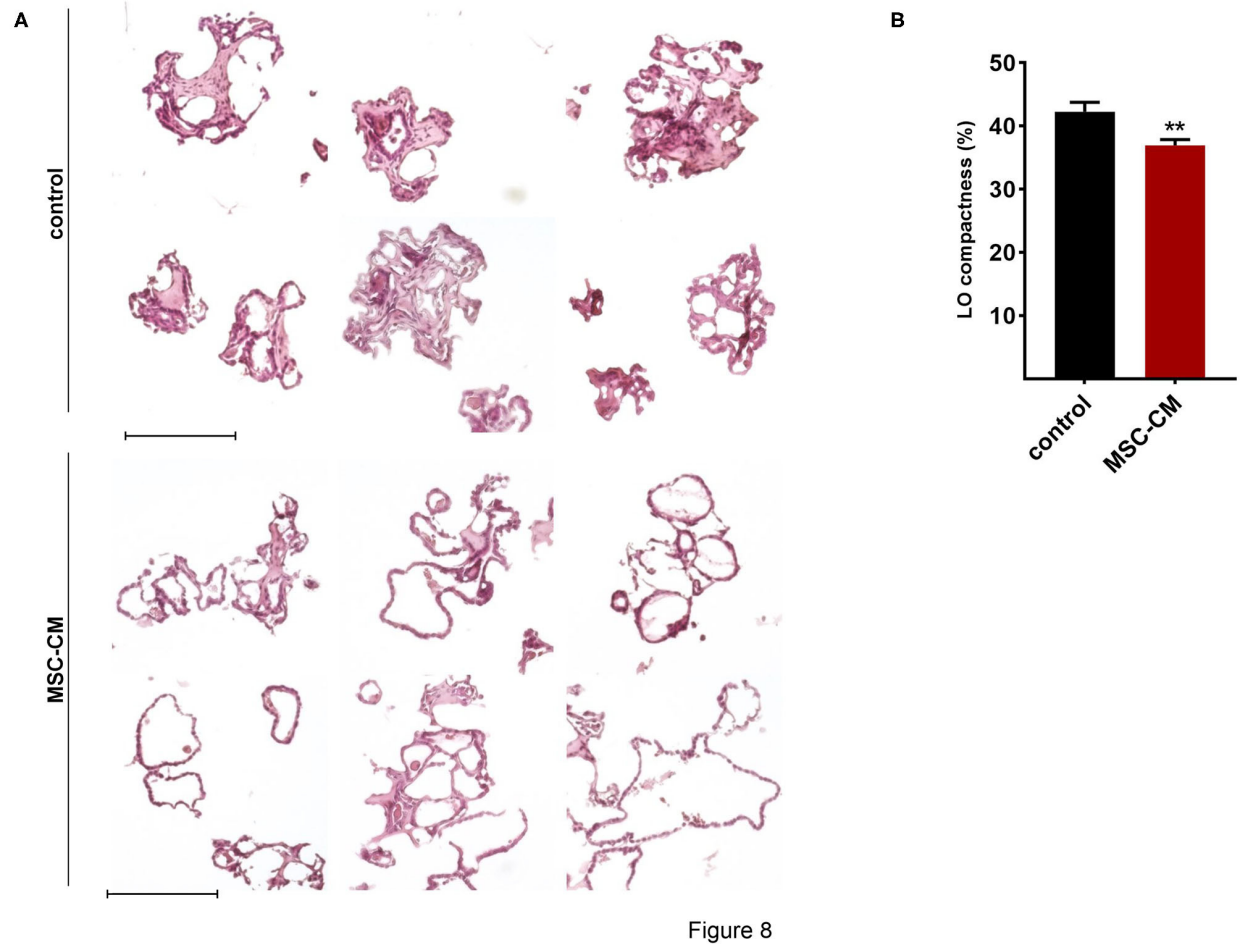
Effects of mesenchymal stem cell-conditioned medium (MSC-CM) on fetal LOs. LOs (15 div) were cultured with MSC-CM for 96 h prior to analysis. **(A)** MSC-CM enhanced lumen formation and thinning of the epithelial layer, in contrast to the denser morphology of control LOs. Scale bar: 200 μm. **(B)** Data are displayed as Mean + SEM. Compactness was higher in control LOs compared to LOs treated with MSC-CM (*n* = 56; ^**^p < 0.01 by *T*-test). div, days *in vitro*.

**Figure 9:**
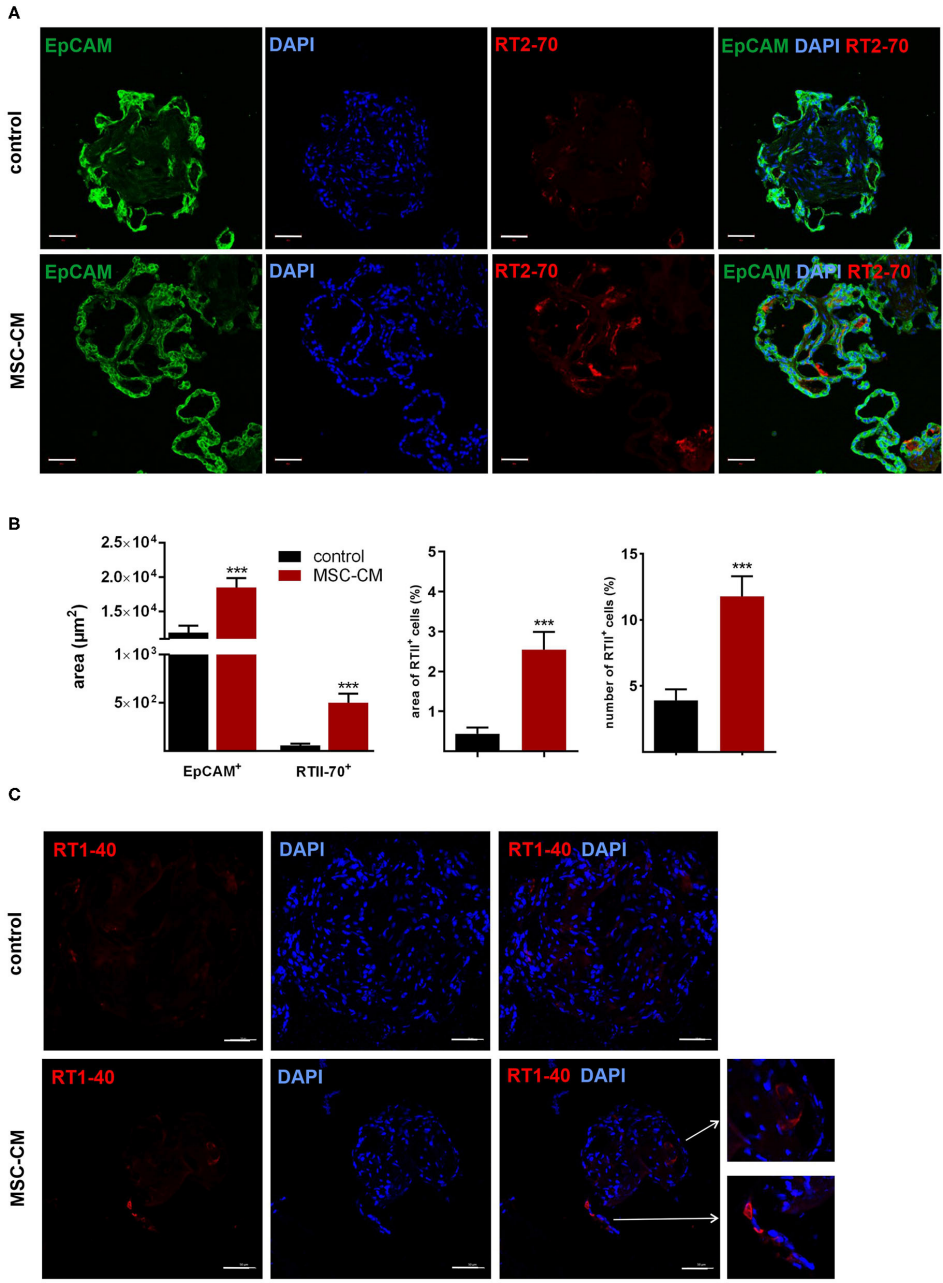
Effects of mesenchymal stem cell-conditioned medium (MSC-CM) on fetal LOs. LOs (15 div) were cultured with MSC-CM for 96 h prior to analysis. Fluorescence images of LO slices were taken by confocal microscopy. **(A)** Organoids were strongly positive for EpCAM expression. Nuclei were stained with DAPI. Some epithelial cells showed luminal expression of the RT2-70 antigen. Scale bar: 50 μm. **(B)** Data are displayed as Mean + SEM. MSC-CM significantly increased the area of EpCAM and RT2-70 antigen expression, as shown by immunofluorescence and the percentage of RT2-70^+^ cell area within the LOs (*n* = 20; ^***^p < 0.001 by *T*-test with Welch's correction). Furthermore, RT2-70^+^ cell numbers were increased by MSC-CM (*n* = 20; ^***^p < 0.001 by Mann-Whitney test). **(C)** The ATI cell marker RT1-40 was observed in some individual cells of the MSC-CM-treated LOs. Scale bar: 50 μm. div, days *in vitro*.

Mesenchymal stem cells (MSCs) have demonstrated therapeutic potential in animal models of neonatal lung disease (21–23) and thus represent a promising future therapeutic approach to alleviate disease burden in preterm infants. In our previous study we determined the paracrine effect of MSCs on lung functional and structural development in FDLE cells and fetal lung explants (19). Herein we aimed at reproducing the reactivity of LOs in comparison to the prior study and to extend the knowledge about cellular effects of MSCs. The LOs (15 div) were cultured with mesenchymal stem cell-conditioned medium (MSC-CM) for 4 days. The respective LO control was incubated with medium used for MSC culture, without MSC conditioning. MSC-CM enhanced lumen formation and thinning of the epithelial layer, in contrast to the denser morphology of control LOs (Figure 8A). This was quantified by the compactness of the LOs, which is defined as the cellular area in proportion to the whole organoid area. Control LOs displayed a higher compactness compared to LOs treated with MSC-CM (*p* < 0.01; Figure 8B). These results show that MSC-CM-treated LOs exhibit a lower cellular packing density and higher intercellular space cavities in contrast to control LOs. Moreover, MSC-CM significantly increased the area of EpCAM and RT2-70 antigen expression, as shown by immunofluorescence and the percentage of RT2-70^+^ cell area within the LOs (*p* < 0.001; Figures 9A,B). This was accompanied by an elevated RT2-70^+^ cell number, which increased from 3.90 ± 3.67% (Mean ± SD) in control LOs to 11.78 ± 6.84% in MSC-CM-treated LOs (*p* < 0.001; Figure 9B). While we could not detect T1α using the antibody RT1-40 in LOs at 15 div, staining of LOs treated with MSC-CM showed at least some RT1-40^+^ ATI cells (Figure 9C). It is open whether this may be due to the four additional days in culture or the change of medium.

## Discussion

The study describes the establishment of fetal rat LOs as a relevant *in vitro* model of fetal lung development that allows scientists to replicate and functionally analyze key elements of lung maturation. For the first time, patch clamp measurement demonstrated single ion channel activity in LOs. The responsiveness of fetal rat LOs to glucocorticoid mimicked the response *in vitro* and *in vivo*, thereby enhancing the relevance of the established model. Furthermore, the response of LOs to MSC-CM demonstrated the convenience of the model to test future therapeutic strategies to enhance maturation of immature lungs.

Even though the first described LOs were created using an undefined cell mix from fetal rat lungs (7), the further development of defined 3D organoid cultures in hydrogels was focusing on mouse and, later, human cells. Defined LOs were successfully generated from mouse and human cells, using primary fetal and adult lung cells as well as pluripotent cells, including embryonic stem cells (ESCs) and induced pluripotent stem cells (iPSCs) (10–12, 24–29). However, the rat is an important animal model and has been widely studied to gain a better understanding of physiological, developmental and pathophysiological mechanisms. Compared to rodent models, the use of human LOs harbors several limitations. Besides the limited availability of fetal lung tissue, its use is highly restricted due to ethical considerations. Similar ethical concerns can be raised for ESCs. ESCs or iPSCs possess great potential to model development and differentiation, but their use is accompanied by the need for a specialized laboratory and technical expertise, the high cost of cell culture, and the long time required for differentiation and production of respective LOs. In contrast, murine LO generation is feasible and can be easily reproduced. Another big obstacle of human fetal lung models and iPSCs is that they cannot be directly compared to the situation *in vivo*, while for rodents a comparison with animal models is possible. This may help to define the possibilities as well as limitations of the different *in vitro* fetal lung models, especially as basis for a future comparison with human fetal lung models. In addition, comparison of *in vivo* validated murine LOs with murine or human LOs from ESCs or iPSCs will show whether LOs from non-lung sources can reflect essential lung functions and key elements of lung maturation.

### Morphological Characterization of Fetal Lung Organoids

During their formation, fetal LOs demonstrated different morphologies of cystic, branched, and mixed appearance. Submerged culture mainly resulted in cystic LOs and cilia activity was observed in some of these cystic LOs. In contrast, ALI culture induced a heterogeneous cystic and branched morphology of LOs. Direct co-culture and interaction between CD31^+^ and FDLE cells was required for LO formation within 24 h, while indirect co-culture delayed the LO formation and reduced the efficacy. Furthermore, direct co-culture with lung mesenchymal cells like fetal lung fibroblasts as well as human CD31^+^ cells (HUVEC) could also support LO formation. Endothelial and mesenchymal cells were highly migratory in Matrigel, while FDLE cells did not show active migration. Direct cell-cell contact between endothelial/mesenchymal cells and FDLE cells facilitated initial cell aggregation required for organoid formation. Thus, the effect of helper cells may not be strictly cell-type specific, but rather based on their migratory capacity, and both endothelial and mesenchymal cells can support organoid growth. In addition, paracrine mechanisms might also be involved, since indirect co-culture with helper cells also resulted in, although delayed, LO formation, which is in contrast to FDLE cell-only culture that lacked LO formation. However, a big advantage of endothelial cells is that their proliferation is not supported under LO culture conditions, while mesenchymal cells proliferate and overgrow the whole cell culture. Notably, all cells in the LOs were positive for EpCAM staining, demonstrating that it consisted only of epithelial cells. Since culture conditions involved a rich serum-containing medium in combination with an ECM, CD31^+^ cells were probably not required as feeder cells, but rather for the 3D assembly of FDLE cells. CD31^+^ cells were highly migratory and provided early tube-like structures. The culture conditions allowed for formation and long-time persistence of vital LOs, showing only a few dead cells in the periphery. Although, these dead cells were not analyzed, the fact that LOs consisted of EpCAM^+^ cells only and CD31^+^ cells were not proliferating, these dead cells could well be leftover CD31^+^ cells. Concerning the maximal size reached at about 15 div, prior studies demonstrated that the ECM environment (Matrigel in our case) imposes solid stress on the growing organoid. Organoids in a mechanically resistant matrix grow until a growth-inhibitory threshold level of solid stress is attained. This is accompanied by an increase of cellular packing density, decreasing apoptosis with no significant changes in proliferation (30). It is therefore assumed that the maximum LO size depends on the mechanical properties of the ECM used.

Staining of LOs demonstrated the presence of EpCAM^+^ cells and cells being also positive for RT2-70 or CC-10, which are markers of ATII and Club cells, respectively. The EpCAM^+^ cells were polarized with the apical side facing the lumen, shown by the apical expression of RT2-70. In contrast to the ATII and Club cell markers, no ATI cells (RT1-40^+^ = T1α) were observed. T1α is expressed throughout lung morphogenesis although on a low and widespread level. T1α mRNA and protein expression increases during late fetal development and is then restricted to ATI cells in the distal epithelium (31, 32). We assume that the lack of RT1-40 staining in our fetal LOscould be based on an immature differentiation state with low T1α expression. Alveolarization start in rodents at postnatal day 4, while our FDLE cells are isolated at the saccular stage of lung development prior to actual development of alveoli. In our research context this immature LO mimics lung immaturity of fetal infants born prior to the start of alveolarization. We assume that the budding branched luminal structures we observe in the LOs possibly represent sacculi, a precursor which correspond to the later alveolar sacculi. In agreement, fetal rat lung slices of the same gestational age were negative for T1α protein staining, while adult rat lung slices were strongly positive for T1α.

### Functional Characterization of Fetal Lung Organoids

Ion channel activity is important for lung growth *in utero* as well as for the perinatal adaptation to air breathing. The analysis of ion channels thus demonstrates a biological function of high physiological relevance with regard to fetal lung maturation. In general, the phenotype of isolated ATII cells under classical culture conditions differs from mature cells *in vivo*. They show differences with regard to cell-cell-communication, expression of tight junctions proteins, barrier function, and general morphology [reviewed by (33)]. Herein we show that fetal LOs represent a physiological model for AFC, as the contributing ion channels can be measured with patch clamp. We demonstrate the presence of ion channels with HSC and NSC channel-like properties in accordance with previous studies of vital lung slices, which showed an average conductance of 8.8 ± 3.2 pS (HSC) and 22.5 ± 6.3 pS (NSC) (34). We observed currents that reverse near 0 mV and exhibit little rectification, which is in contrast to the current-voltage relationship for HSC and NSC channels from ATII cells in primary culture (35). Therein, HSC channels strongly rectify and reverse at high depolarizing potentials, and NSC channels reverse at ~+40 mV (35). High K^+^ concentration in our bathing solution is supposed to depolarize the cell and to establish a resting membrane potential near 0 mV. Thereby the holding potential installed at the pipette should represent the actual patch potential. By their nature NSC channels must reverse at a patch potential of 0 mV, which means that the observed reversal potential is representative of the membrane potential. Thus, in primary ATII cells the membrane potential is ~-40 mV, while in our depolarized LOs the membrane potential must be close to 0 mV, equal to the observed reverse potential. The same was reported for vital lung slices, which demonstrated HSC and NSC channels that reverse near 0 mV and HSC channels exhibiting little rectification (35, 36). Possible causes for the differences between cultured primary ATII cells and lung slices have been discussed in detail by the authors (35). It further underlines that LOs more closely represent vital lung tissue in contrast to primary ATII cells, which is important for studying ion transport in alveolar cells. Furthermore, anion channels with properties like ORCC were observed, which possibly contribute to balancing the electroneutrality of Na^+^ transport in ATII cells. In our study, we did not aim at thoroughly depicting ion channel activities in fetal LOs, but to demonstrate the biological function and applicability of the model system. According to our observations, fetal LOs constitute a physiological model system to study the single channel activity of the (fetal) alveolar epithelia.

### The Stimulating Effects of Dexamethasone and MSCs

After the characterization of LO function, we determined their responsiveness to established lung maturation-inducing hormones. Antenatal glucocorticoids accelerate late-gestation lung maturation in low doses by enhancing surfactant synthesis, increasing the volume density of ATII cells and upregulating AFC (37–39). Mice lacking intracellular glucocorticoid receptors died of respiratory failure shortly after birth (40). Their lung development was retarded, accompanied by a reduction of *ENaC* mRNA levels in total lung RNA (40). Several studies demonstrated the stimulation of *ENaC subunit* expression by glucocorticoids (13, 41). In accordance, dexamethasone strongly increased mRNA expression of all *ENaC subunits* and that of the rate-limiting *Na,K-ATPase* β_1_-*subunit* in fetal LO. In contrast, *CFTR* mRNA expression was reduced by dexamethasone as previously shown in FDLE and human bronchial submucosal gland-derived Calu-3 cells (42, 43). Furthermore, dexamethasone stimulated mRNA expression of *Sftpb* and *Sftpc*, confirming the surfactant synthesis-stimulating effect. We complemented the analyses of mRNA expression in LOs with measurements done in our FDLE cell model. The comparison showed a similar increase of *ENaC* and *Na,K-ATPase* mRNA expression induced by dexamethasone. Furthermore, the elevated Na^+^ transport and ENaC activity stimulated by dexamethasone was shown in Ussing chamber measurements, demonstrating the relevance of elevated mRNA expression for channel activity. These results confirm the validity of our fetal LO model reproducing the response to glucocorticoids seen *in vitro* and *in vivo*. Furthermore, dexamethasone increased the area of EpCAM and RT2-70 antigen expression, thereby underlining the stimulating effect of glucocorticoids on alveolar differentiation.

Regarding the immature state of lungs from preterm infants, developing and testing new therapeutic strategies is of high clinical relevance. Due to the immunomodulatory and regenerative potential of MSCs, MSC-based cell therapies represent an interesting therapeutic strategy to enhance lung maturation (23). The therapeutic potential of MSCs is mainly attributed to paracrine effects. In accordance, MSC-CM affected LO morphology with an enhanced lumen formation as well as an increased expression of EpCAM and RT2-70. The results showed that up to 15% of the cells expressed the RT2-70 antigen. This is close to the situation *in vivo*, where ATII cells comprise ~15% of all lung cells, but cover only ~2–5% of the internal surface area (44). These results suggest an enhanced maturation of LOs induced by MSC-CM, which is in line with a prior study of our group (19). Therein we showed that MSC-CM strongly stimulated functional and structural maturation of fetal lungs. Fetal lung explant growth and branching as well as surfactant protein mRNA expression were enhanced by MSC-CM (19). Furthermore, MSC-CM strongly increased the activity and mRNA expression of *ENaC* and the *Na,K-ATPase* in FDLE cells (19). These effects were at least partially mediated by the PI3-K/AKT (phosphoinositide 3-kinase/protein kinase B) and Rac1 (Ras-related C3 botulinum toxin substrate 1) signaling pathways (19). In agreement, we demonstrated changes in structural maturation in LOs stimulated with MSC-CM, as shown by reduced compactness, increased ATII cell number and area, and first detection of ATI cells. Therefore, results observed in fetal rat lung explants as well as primary FDLE culture were reproduced in fetal LOs, confirming its relevance as a functional *in vitro* model, which can be used to study novel therapeutic developments.

### Limitations and Outlook

There are also several limitations of our fetal LOs as an *in vitro* model. An important aspect to consider is the undefined culture condition, which may affect research focused on differentiation and signaling pathways. The culture conditions applied in this study uses FCS and Matrigel, both complex, undefined, and batch-to-batch varying solutions isolated from primary tissue sources of different species, supplying growth factors or basement membrane proteins. Adapting the culture conditions of LOs in the future will possibly enable the generation of more complex co-culture systems to study direct and indirect cellular interactions.

In general, a detailed morphological analysis of fetal rat lung cells and tissues was challenging, since antibodies specific for rat lung tissue are rare. Furthermore, differences in antibody binding between fetal and adult lung tissue were observed and organ specific expression was also seen. Although several studies have shown that the monoclonal antibody RT2-70 is specific for the apical surfaces of rat ATII cells, the respective protein is largely unknown. It is therefore also unknown at which maturational state an ATII cell expresses this unknown protein and whether a lack of expression rules out an ATII cell identity. The target protein is expressed only at the apical membrane compartment, hence only a small area of the cell is stained, but most luminally located cells expressed the RT2-70 antigen. This is in contrast to the EpCAM staining, which can be found on the complete cell surface. Correlating the area positive for EpCAM to the whole LO area in comparison to the area positive for RT2-70 does not reflect the number of ATII cells and might underestimate the amount of ATII cells in our LOs. Despite these limitations FDLE cells are a physiologically well-characterized *in vitro* model that has been studied with Ussing chambers and patch clamp in addition to mRNA expression analyses. We have determined the effect of glucocorticoids, female and male sex hormones, insulin and many other factors on the maturity of FDLE cells in prior studies (45–49), which enabled us to compare the different *in vitro* fetal lung models and to evaluate if the generated 3D fetal LOs represent a novel *in vitro* model suitable for studies addressing developmental or therapeutic approaches. The FDLE cells are derived from fetal rat pups 24–48 h prior to birth. Studies showed that fetal rat pups born 24 h prior to term birth experience respiratory distress due to structural and functional lung immaturity, reflected in a survival rate of only 6% by 36 h after delivery if the pups were placed in air, which increased to 47% when they were placed in >95% oxygen (4). The authors concluded that the preterm rat is a suitable model for studies of acute and chronic neonatal lung disease, as structural and functional lung immaturity is a major risk factor for pulmonary complications. Due to this and other reports we believe that our model is suitable to study immaturity-associated complications arising from preterm birth.

While the CD31^+^ cells used in this study were required for an efficient LO formation, their role and interactions seemed to be limited to the first days. It would be interesting to analyze how a combined co-culture of endothelial and mesenchymal cells may further enhance LO differentiation, especially with defined medium and ECM conditions. This could also provide an *in vitro* model to study the effects of inflammation on lung development by either applying proinflammatory stimuli or using a direct or indirect co-culture approach with cells of the innate immune system. Another aspect, which should be considered to enhance LOs as *in vitro* lung model, is the biophysical property of the environment, although this may be more technically challenging to adapt. This includes the defined elastic moduli of hydrogels, the oxygen concentration to present hyperoxia or hypoxia as well as biomechanical stimuli and stress induction by periodical stretching or acute pressure. Despite these limitations and currently unsolved challenges, LOs provide a fast and easy *in vitro* model that allows a faster screening procedure compared to animal models, while also providing a higher biological relevance compared to classical cell culture. LOs will allow for a broad range of manipulation and can be adjusted to the study needs like changing signal pathways using agonists or antagonists, adaption of the physical and chemical properties of the ECM, genetic manipulation of the cells used for LO formation, and/or co-culture with different cell types or pathogens. This could lead to more complex models with multiple and/or sequential impacts to recapitulate fetal and/or newborn lung injuries.

## Conclusion

In conclusion, LOs generated from FDLE cells represent a fetal lung model that replicates key biological lung functions essential for lung maturation. In detail, the fetal LOs demonstrated the development of fetal lung alveoli, the expression of surfactant proteins, and most importantly the expression and electrophysiological activity of ion channels. For the first time electrophysiological analysis by patch clamp allowed the single cell measurement of ion channel activity in LOs. Furthermore, fetal LOs showed functional responsiveness to glucocorticoids and MSCs. The main goal was to develop an immature LO model to enable the study of maturation and how this can be enhanced to benefit preterm infants in the future. Thus, fetal LOs demonstrated the convenience of the model to test and establish new therapeutic strategies.

## Data Availability Statement

The original contributions presented in the study are included in the article/Supplementary Materials, further inquiries can be directed to the corresponding author.

## Ethics Statement

The animal study was reviewed and approved by Landesdirektion Leipzig.

## Author Contributions

ML and CF: conceptualization, formal analysis, funding acquisition, resources, supervision, and writing-original draft. ML, SP, TP, and CF: data curation, investigation, and methodology. ML, SP, and CF: validation. All authors writing-review and editing.

## Conflict of Interest

The authors declare that the research was conducted in the absence of any commercial or financial relationships that could be construed as a potential conflict of interest.

## Publisher's Note

All claims expressed in this article are solely those of the authors and do not necessarily represent those of their affiliated organizations, or those of the publisher, the editors and the reviewers. Any product that may be evaluated in this article, or claim that may be made by its manufacturer, is not guaranteed or endorsed by the publisher.
