# Supplementary figures and images for: Development and Functional Characterization of Fetal Lung Organoids
Source: Front Med (Lausanne). 2021 Sep 6;8:678438. doi: 10.3389/fmed.2021.678438 (PMC8450364; doi:10.3389/fmed.2021.678438)

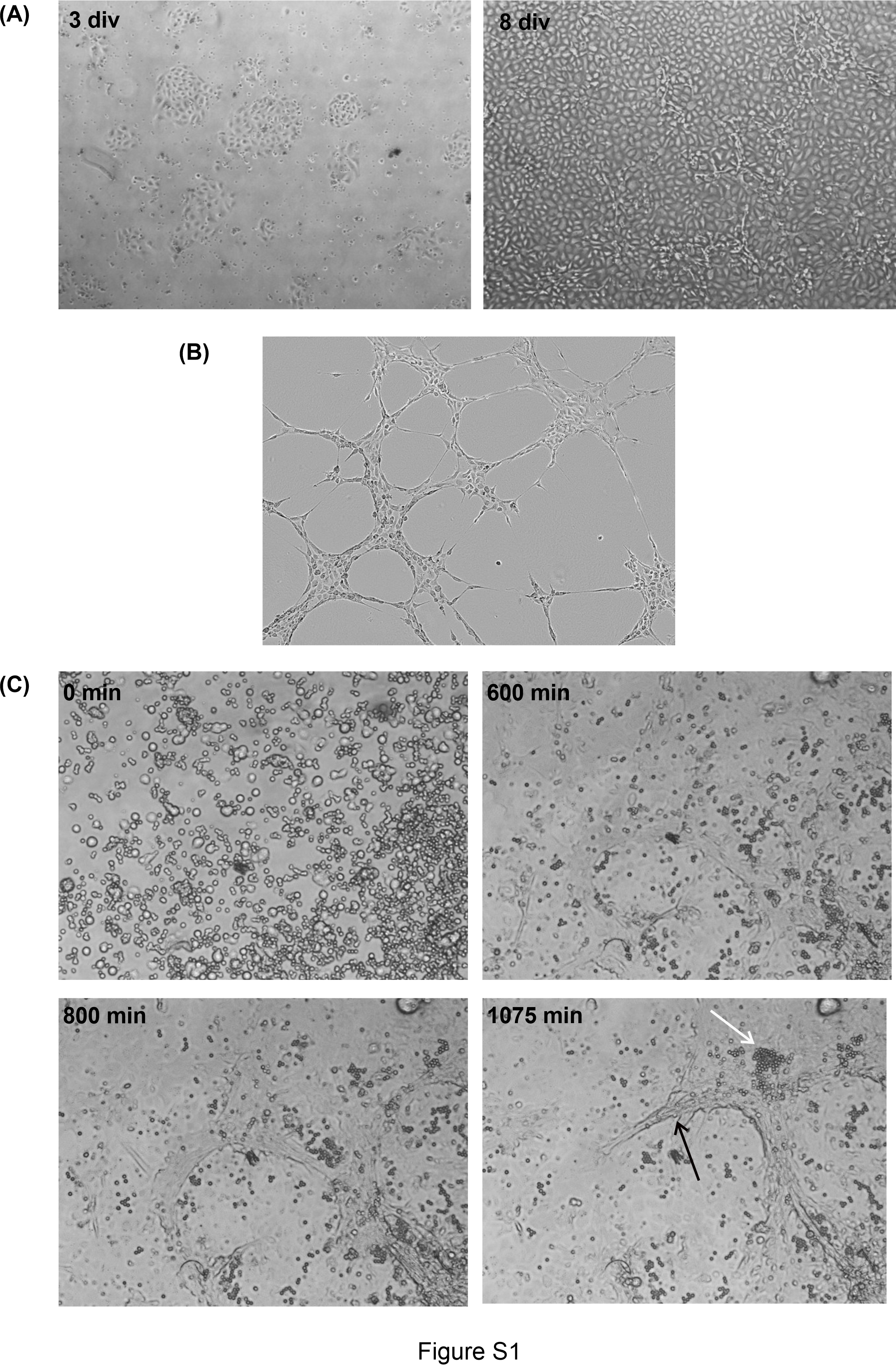

Supplement: Supplementary file 1 [file Image_1.JPEG]

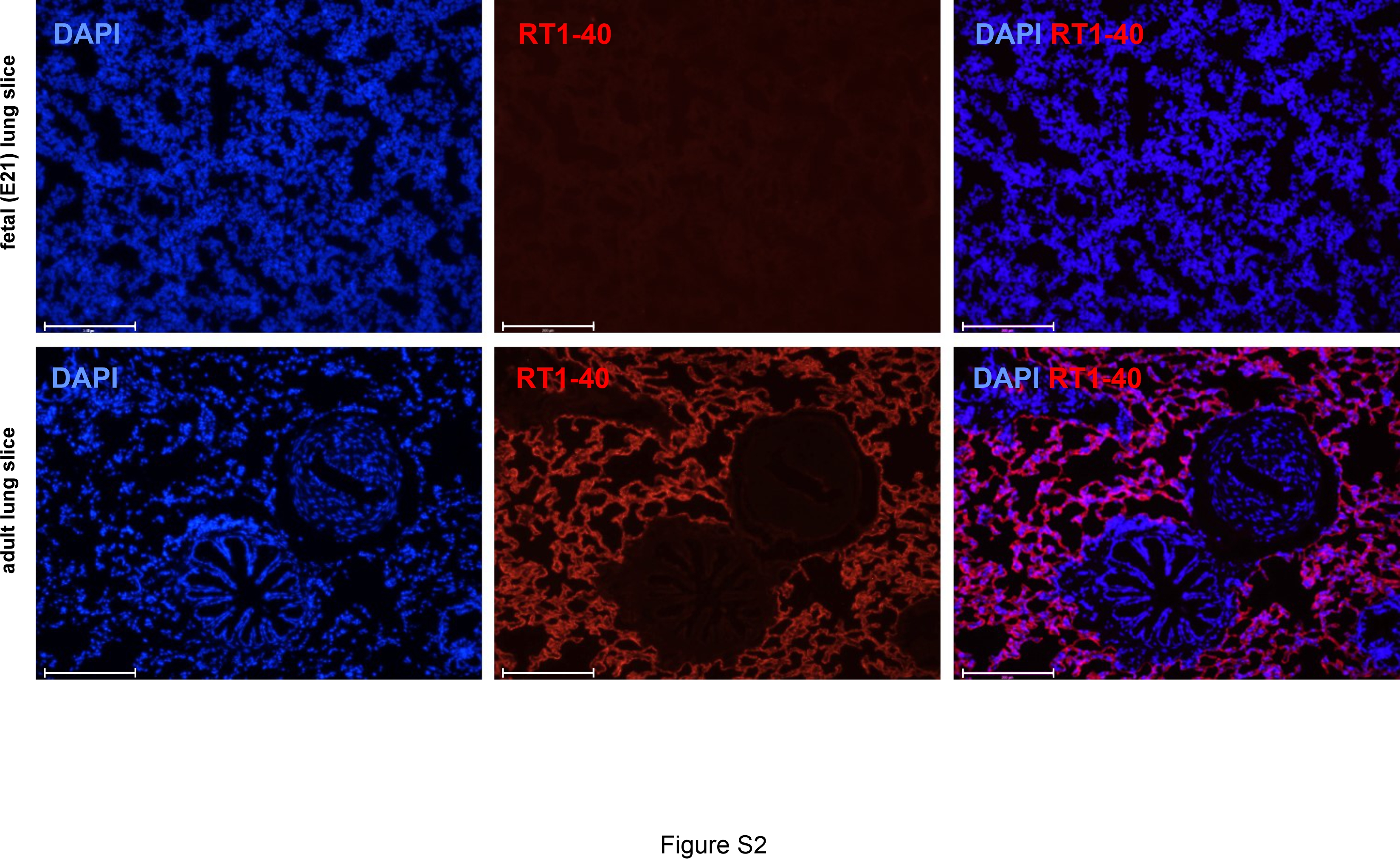

Supplement: Supplementary file 2 [file Image_2.JPEG]

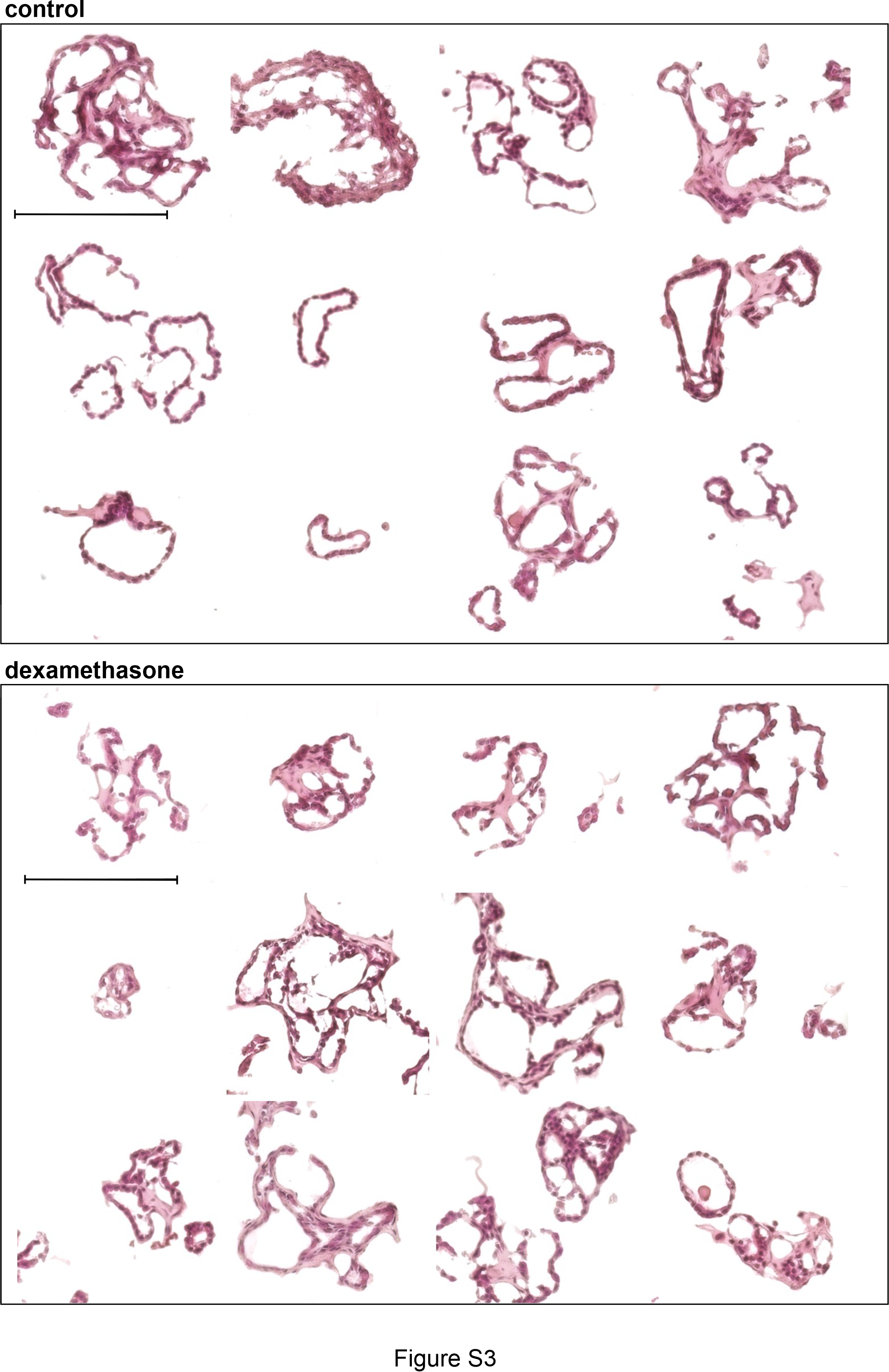

Supplement: Supplementary file 3 [file Image_3.JPEG]
