# Supplementary material for: Development and Functional Characterization of Fetal Lung Organoids
Source: Front Med (Lausanne). 2021 Sep 6;8:678438. doi: 10.3389/fmed.2021.678438 (PMC8450364; doi:10.3389/fmed.2021.678438)
Supplement: Supplementary file 4 [file Data_Sheet_1.DOCX]

**Supplemental figure legends**

**Supplemental figure 1: Fetal lung endothelial cells.** Cell culture microscopic images at 100-fold magnification. **(A)** Morphology of CD31^+^ cells from fetal rat lungs cultured on gelatine-coated cell culture plastic with ECGS medium. Single cell colonies after isolation of CD31^+^ cells plated on gelatine-coated cell culture plastic (3 div). **(B)** Confluent monolayer of CD31^+^ cells plated on gelatine-coated cell culture plastic (8 div). **(C)** Tube formation of CD31^+^ cells plated on Matrigel (GFR) coated cell culture plastic after 24 h. **(D)** Live cell imaging of LO formation during the first 17 h. Freshly isolated FDLE cells were mixed with CD31^+^ cells and combined with Matrigel (GFR) in a 24-well plate. Images were taken every 5 min. FDLE cells aggregated along CD31^+^ cells (white arrow). CD31^+^ cells formed tube like structures inside the Matrigel (black arrow). div: days *in vitro*.

**Supplemental figure 2: Fetal and adult rat lung slices.** Fluorescence images of fetal (E21) rat lung slices (upper panel) and adult rat lung slices (lower panel). Nuclei were stained with DAPI. Expression of the ATI cell marker RT1-40 was only observed in adult rat lung slices. Scale bar: 200 µm.

**Supplemental figure 3: Effect of dexamethasone on fetal LOs.** LOs (15 div) were stimulated with dexamethasone (100 nM) for 48 h. No obvious morphological alterations were induced by dexamethasone compared to control LOs. Scale bar: 200 µm.
